# Endurance and Gait Speed Associations with Incident Dementia in Older Adults: The ARIC Study

**DOI:** 10.1093/geroni/igaf122.2904

**Published:** 2025-12-31

**Authors:** Jamesia Kenney, Hunter Sylvester, Michael Griswold, Rebecca Gottesman, David Knopman, Kelley Pettee Gabriel, Kevin Sullivan, B Gwen Windham

**Affiliations:** University of Mississippi Medical Center, Jackson, Mississippi, United States; University of Mississippi Medical Center, Jackson, Mississippi, United States; University of Mississippi Medical Center - The MIND Center, Jackson, Mississippi, United States; National Institutes of Health, Bethesda, Maryland, United States; Mayo Clinic Department of Neurology, Rochester, Minnesota, United States; The University of Alabama at Birmingham, Birmingham, Alabama, United States; University of Mississippi Medical Center - The MIND Center, Jackson, Mississippi, United States; University of Mississippi Medical Center - The MIND Center, Jackson, Mississippi, United States

## Abstract

Faster gait speed and better endurance have been cross-sectionally and jointly associated with a lower likelihood of dementia. However, gait speed associations plateau above 1m/s, suggesting gait speed may be less predictive at near normal speeds. This study examined prospective associations of gait speed, endurance, and moderating effects of endurance on gait speed associations with incident dementia among ARIC participants without dementia at Visit 6 (2016-2017) who completed assessments of endurance ( 2- Minute Walk (2MW), meters) and usual-pace 4-meter gait speed (m/s) (n = 2767, mean age 78+/- 4.3 years, 43% male, 18% Black). Dementia was adjudicated using neuropsychological tests, functional assessments, informant interviews, and medical/vital record surveillance over a median of 3.3 years follow-up. Cox regression models estimated the hazard ratio (HR, 95% CI) for dementia, incorporating gait speed, 2MW, gait speed-by-2MW interaction, demographic, and cardiovascular factors. In separate models, each standard deviation higher 2MW [HR = 0.62, (0.50,0.76)] and faster gait speed [HR = 0.63, (0.50,0.81)] were associated with lower incident dementia risk. However, when jointly adjusting for 2MW and gait speed, higher 2MW was associated with a 32% lower incident dementia risk [HR = 0.68,(0.51, 0.90)], but faster gait speed was not statistically associated with incident dementia [HR = 0.85, (0.62,1.16)]. Moderating effects were not supported (gait speed-x-2MW interaction, p = 0.14). Although both endurance and gait speed predict dementia, endurance may be more informative across the gait speed spectrum, whereas gait speed may be more feasible for clinical settings. Longer follow-up and more dementia events may better inform differential utilities of gait speed and endurance measures.

